# Surgical assessment: measuring unobserved health

**DOI:** 10.1186/1471-2482-15-4

**Published:** 2015-01-15

**Authors:** Trafford Crump, Kevin Wing, Nick Bansback, Jason M Sutherland

**Affiliations:** Department of Pediatrics, Medical College of Wisconsin, Center for Clinical Effectiveness Research, Children’s Hospital of Wisconsin, Milwaukee, WI USA; Department of Orthopaedic Surgery, School of Medicine, University of British Columbia, Vancouver, British Columbia Canada; School of Population and Public Health, University of British Columbia, Vancouver, British Columbia Canada; Centre for Health Services and Policy Research, School of Population and Public Health, University of British Columbia, Vancouver, British Columbia Canada

**Keywords:** Waiting lists, Access to health care, Referral and consultation, Secondary care, Elective surgical procedures, Health status, Quality of life, Health surveys, Longitudinal survey

## Abstract

**Background:**

The federal and provincial governments in Canada have invested an enormous amount of resources to measure, report and reduce surgical wait times. Yet these measures under-report the wait period that patients’ actually experience, because they do not capture the length of time a patient spends waiting to see the surgeon for a surgical assessment. This unmeasured time is referred to as the “wait one” (W1). Little is known about W1 and the effects that this has on patients’ health. Similarly, it is not understood whether patients waiting for surgical assessment actually want or need surgery. Existing administrative and clinical dataset do not capture information on health and decision-making while the patient is waiting for care form a specialist. The objective of this proposed study is to understand the impact that W1 for elective surgeries has on the health of patients and to determine whether this time can be reduced.

**Methods/Design:**

A prospective survey design will be used to measure the health of patients waiting for surgical assessment. Working with the support of the surgical specialities in Vancouver Coastal Health, we will survey patients immediately after being referred for surgical assessment, and every four months thereafter, until they are seen by the surgeon.

Validated survey instruments will be used, including: generic and condition-specific health status questionnaires, pain and depression assessments. Other factors that will be measured include: patients’ knowledge about their condition, and their desired autonomy in the decision making process. We have piloted data collection in one surgical specialty in order to demonstrate feasibility.

**Discussion:**

The results from this study will be used to quantify changes in patients’ health while they wait for surgical assessment. Based on this, policy- and decision-makers could design care interventions during W1, aimed at mitigating any negative health consequences associated with waiting. The results from this study will also be used to better understand whether there are factors that predict patients’ desire to proceed to surgery. These could be used to guide future research into experimenting with interventions to minimize inappropriate referrals and where they are best targeted.

## Background

Patients typically do not have immediate access to elective surgery in Canada. Instead most patients have to wait, and that wait time is comparatively longer than wait times in other developed countries. Canada ranks as the poorest performer among a sample of 11 industrialized countries in access to hospital care for adult patients; 25% of patients wait more than four months for elective surgery and 41% of patients wait more than two months to see a specialist [1]. For some patients, this wait time impacts their quality of life [2, 3].

Currently, provinces report surgical wait times starting from when patients are placed on the surgical wait list until the time of surgery. This snapshot provides limited insight into the actual duration of a patient’s wait. There is often a lengthy wait from the time a referral to a specialist is made until the time of the initial surgical assessment. This time period, which we refer to as “Wait One” (W1), usually goes unmeasured, but represents an important part of a patient’s care experience.

Little is known about patients’ health while waiting for surgical assessment. Surgeons and referring physicians report that this wait time can be very lengthy and causes a significant disruption in patient’s care [4]. It is not clear how many patients waiting for surgical assessment actually wish to proceed to surgery. A study of patients’ treatment decisions after seeing an orthopaedic surgeon in Ontario reported that 79.3% of patients did not get surgery, indicating that not all referrals may be appropriate for surgical consultation [5]. The study’s authors posited that many referrals to orthopaedics are for specialist input into the management of the underlying condition, and suggest that further research is needed to better understand the kind of patients being referred to surgical specialties and the reasons for those referrals.

### Objective

The objective of this proposed study is to understand the impact that W1 for elective surgeries has on the health of patients and to determine whether this time can be reduced. This objective will be met by answering four research questions:

1. What is the length of time patients spend waiting for their initial surgical assessment?
2. What is the change in health status that patients experience while waiting for their initial surgical assessment?
3. What are patient factors associated with the decision to proceed with surgery?
4. Does self-reported information help to discriminate between warranted and unwarranted referrals?

This study is being undertaken in collaboration with several surgical groups practicing in Vancouver Coastal Health (VCH) authority. This research team has strong historical relationships with many specialty surgical groups practicing in VCH’s six hospitals, the VCH executive team and the British Columbia (BC) Ministry of Health, ensuring the study’s feasibility and maximizing the likelihood that the findings from this study will be incorporated into decision- and policy-making processes.

### A brief review of the literature

#### How long do patients wait for elective surgical assessment?

There are no population-based provincial initiatives that collect patients’ health information while they are waiting for surgical assessment in Canada. Published literature on the period between referral to, and assessment by, a surgical specialty are limited to individual specialties reported at a single instance in time – nor do they study health outcomes or the decision-making processes. In 2005, the Canadian Association of Gastroenterology reported that the median wait time to see one of their specialists for inflammatory bowel disease (which has a two-week target) was 101 days (inter-quartile range 35–209 days) [6]. In 2006, the Canadian Institute for Health Information (CIHI) reported that for patients having hip or knee replacement surgery, 30% of their overall wait was for the initial assessment [7]. Another study of wait times from referral to surgical assessment for hip and knee arthroplasty in Alberta determined that the mean wait time for this period ranged from 51 to 139 days [8]. The study identified that approximately 40-80% of the wait time for patients was during the wait for surgical assessment [8]. One study in Ontario used general practitioner (GP) electronic medical records to track wait times to see a specialist over a five year period [9]. The study found high variability in wait times based on specialty and GP practices, men had a shorter median wait then women (51 and 55 days, respectively) and younger patients had the shortest median wait at 45 days [9].

#### What are the consequences of waiting for elective surgical assessment on patients’ health?

There is an absence of comprehensive data currently available regarding the effects of waiting for elective surgical assessment on patients’ health. This information is crucial, since delays in access to healthcare services for other, non-elective conditions, have been shown to affect the trajectory of care. Research conducted by Prentice and Pizer studying wait times in the Veterans Administration in the United States observed that delays in accessing outpatient services for ambulatory care sensitive conditions, such as surgical assessment, significantly increased the odds of being hospitalized if these delays were over 29 days; if over 31 days, the odds of mortality increased [2, 3].

### Who is waiting for elective surgical assessment?

Critically, there is not much information regarding whether patients waiting for elective surgical assessment really need – or want – surgery. A study in Queensland (Australia) evaluated the effect of implementing a GP referral system aimed at addressing the high wait times for non-urgent specialist appointments [10]. Patients on a wait list who were identified as having long waits were sent letters offering two options: to indicate that the appointment with a specialist for surgical consult was no longer necessary or to update their referral [10]. In the initial stage, 872 patients who had long waits for orthopaedic surgeries were identified and 101 responded, and only 16 of those patients proceeded to surgery [10]. In an expanded program, over 6,885 patients waiting for multiple specialities were contacted. Of these, 633 responded and 197 required surgery [10]. This Australian study helps to underscore that there are a number of patients on the wait list for surgical assessment that may not choose to be, or remain, there.

Taken together, these studies paint a picture of a significant gap in our understanding of the nature of, and changes in, patients’ health and decision making during W1. Addressing this gap in knowledge would provide invaluable insight to patients, clinicians, regional health authorities who manage access to surgical resources and government stakeholders who are ultimately responsible for ensuring effective and efficient use of healthcare spending.

### Conceptual framework

The conceptual basis for this proposal is adapted from a taxonomy proposed by Wennberg et al., in which the utilization of healthcare is classified into one of three categories: effective care, preference-sensitive care and supply-sensitive care [11]. Effective care refers to those clinical scenarios that have an efficacious clinical pathway defined by medical evidence, such as hip fracture repair or appendectomies. This is generally not so for elective surgical care targeted in this study, thus we focus on the latter two categories.

Preference-sensitive care is care for conditions where there is more than one option for treatment and for which the scientific evidence regarding the effectiveness of these options is equivocal [12]. Preference-sensitive care is similar to elective surgical care, where surgery may be one of several treatment options available to the patient. For example, with knee osteoarthritis several non-surgical treatments are available, including weight loss, exercise or steroid treatment. Surgical options for treatment include osteotomy and partial or total knee replacement [13].

In order to provide high quality preference-sensitive care, physicians must take into account a patient’s preference for care and their health goals [12]. This requires that patients be fully informed and have confidence in their decision regarding the treatment choice that best meets their care needs. The quality of referrals for preference-sensitive conditions depends on a patient’s understanding of treatment options, the potential risks for harms and benefits associated with those options and on patient confidence in their decision to have surgery [12]. We define quality referrals as “appropriate”, and no further policy interventions are necessary. Inappropriate referrals do not follow this process.

Supply-sensitive care is care that is based on a local health system’s availability of resources rather than on clinical evidence or a patient’s preference for treatment. Referrals made in this context imply that decisions regarding surgical procedures are based on maximizing available capacity (e.g. operating room time) instead of providing the best care for patients [11, 14]. We consider referrals that do not match patients’ preferences or that are made because of the availability of local surgical resources to be “inappropriate”.

Identifying health system, patient and referring physician characteristics associated with inappropriate referrals for preference- and supply-sensitive surgical care is the basis for this proposed study. Inappropriate referrals to surgical assessment require intervention for two reasons. First, they lead to patients’ unnecessarily occupying space on wait lists for surgical assessment at the expense of patients with appropriate referrals for surgery. Second, inappropriate referrals could be deferred to patient education interventions or active surveillance programs instead of consuming the time and resources of specialists and scarce hospital resources.

**Figure 1:**
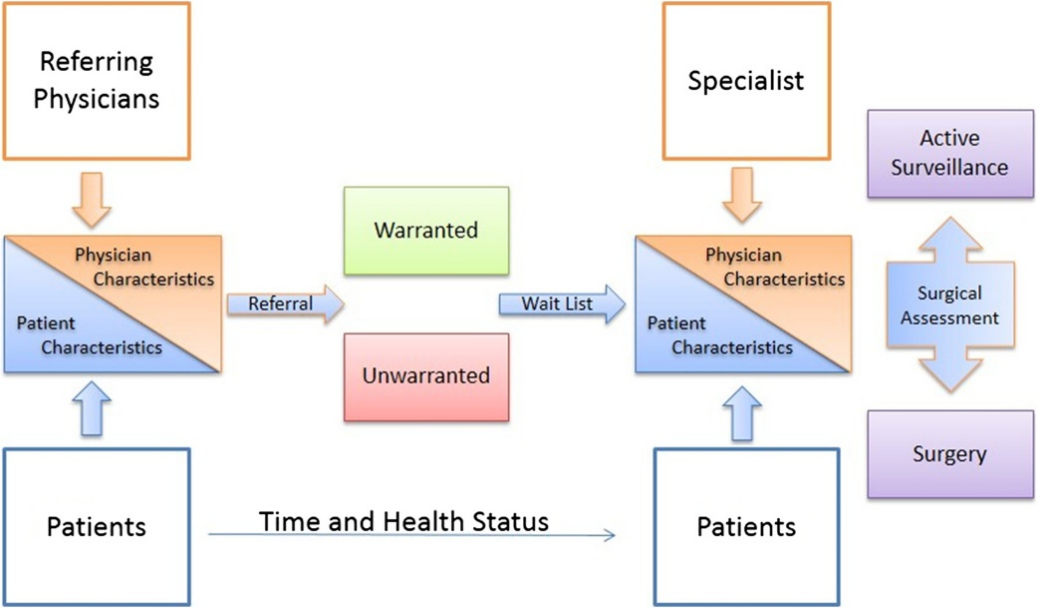
**Conceptual model for the collection and measurement of patients; health data while waiting for surgical assessment.**

The interaction between patients and physicians during the decision to be referred for surgery is governed by their relationship as illustrated on the left half of Figure 1. Referring physicians are often GPs, but may also be other specialists, either within or outside the speciality to which the patient is being referred for surgery. The referring physician is likely familiar with the patient’s medical history, has an established relationship with the patient and may be aware of that patient’s specific health goals or concerns.

Patients’ health goals, well-being, and knowledge level will be factors in their decision to be referred to surgery. Although not all patients wish to be involved in the treatment decision-making process, those that are report greater quality-of-life scores and less regret about their treatment [15, 16]. To understand the role these factors play in referrals and decision to proceed with surgery, this proposed study will measure health, functional well-being and knowledge level at the time of referral for surgery. These measures will be regularly retaken over the course of the W1 period in order to track any changes and their potential impact on a patient’s decision to proceed with surgery.

The interaction between the patient and the surgeon in the decision to proceed with surgical treatment is illustrated on the right half of Figure 1. The patient’s measured level of health, knowledge regarding their condition, and decision confidence may have changed while waiting, thus affecting their decision to proceed with surgical treatment. Using statistical models for repeated measures, we will incorporate changes in patients’ level of health, knowledge regarding their condition and decision confidence over time, and its potential effect on surgical treatment.

Surgeons also play a role in the decision of patients to proceed to surgical treatment. Surgeon effects will be included in our statistical models to reflect treatment preferences, as well as age and utilization profiles. The end point of the study is, based on the surgical assessment, the patient’s decision to proceed with surgical treatment or not. The modalities of treatment prior to referral to surgical assessment are outside of the scope of this study and will be pursued in the future.

## Methods/Design

To answer the research questions in this proposed study, we will use a prospective longitudinal survey design. Study participants will be surveyed at regular intervals from the time they are referred for an elective surgical procedure until the time of their initial surgical consultation. For our purposes, this design is superior to cross-sectional surveys, where patients are asked retrospectively of their experiences. Retrospective studies are subject to recall bias and patients rate their health state higher as they adjust to limitations over time [17].

### Target population

Our sampling frame is those patients newly referred to a surgeon in one of the surgical specialties identified in Table 1 who are practicing in Vancouver, BC. The study will recruit patients referred for an elective surgical procedure from either primary care providers or from other specialties (i.e. cross-referrals). These surgical specialties have been selected for two reasons. First, they represent elective surgical procedures that can be considered preference-sensitive and non-surgical treatment options may be available. Second, we have pre-established relationships with surgeons in these surgical specialties through other studies. While there may be selection bias amongst those specialities that have agreed to participate, there is no reason to believe that patients of these surgeons are systematically different from the population as a whole.

**Table 1:** **Procedures of interest by surgical specialty**

General surgery	Orthopedics	Plastic surgery	Urology
Laproscopic cholecystectomy	Elbow reconstruction	Breast reconstruction	Penile prosthesis
Femoral hernia repair	Knee ACL reconstruction	Mammoplasty reduction	Bladder suspension sling
Ventral hernia repair	Shoulder rotator cuff repair	Abdominoplasty	Urinary artificial sphincters
Inguinal hernia repair	Ankle arthritis	Hand tendon repair	Transurethral resection of the prostate
Gastrointestinal bypass	Bunion repair	Mandibular fracture	
Hemorrhoidectomies			

These surgeons have agreed to share their referral data with the study team. Patients will be contacted by the research team once the referral is received by the surgeon’s office. Though there will be a gap between the patient’s visit to the referring physician and the receipt of the referral by the surgeon’s office, we expect this gap to be less than seven days and to have a negligible effect on the study’s data.

### Sampling inclusion / Exclusion criteria

All patients who have been referred for an elective surgical procedure in Table 1, who are over the age of 18, and who are able to provide verbal and written consent in English will be recruited for the study. Patients will be excluded if they are facility-bound, are unable or unwilling to provide consent or if they demonstrate signs of severe depression or suicidal tendencies.

### Recruitment strategy

When a referral is received by the surgeon’s office, the patient will be asked by the office if they wish to be involved in this study. Patients that are interested will have their contact information securely transferred to a member of the study team. This team member will contact the patient over the telephone, explain the study, emphasize that it will have no impact on their wait time to see the surgeon and obtain verbal consent. Patients that provide verbal consent will be mailed a written informed consent with their initial survey.

### Survey

An initial survey package will be sent to participants and will include: 1) an information brochure outlining the study, 2) an informed consent form to be signed (in duplicate, so the participant may keep a copy), 3) contextual questions regarding their socio-demographic status [18] and most common co-morbidities and 4) questions reading their health status, knowledge about their condition and their decisional conflict (see Measures section below). Participants may opt out of any question(s) they are not comfortable answering. A self-addressed stamped envelope will be included in the package.

### Data collection procedures

The initial survey will be mailed as soon as verbal consent is received, within a week of the referral being received by the surgeon’s office. Subsequent surveys will be mailed every four months until the participant has their consultation with a surgeon. The last point of survey will be just before the surgical assessment. Patients may have different numbers of survey points because the WI period varies by speciality and surgeon. We will identify which patients progress to surgery with the assistance of the surgeon’s office.

### Survey management

A survey management database will be created for the purpose of organizing and coordinating the inbound and outbound survey packages. To cut down on labour needs, the data entry process will be automated by designing and printing surveys on scannable forms. Only the study’s principal investigator, survey coordinator and data entry staff will have access to the database. The statistician will have access to de-identified data.

### Participant retention

To enhance participant retention, reminders will be mailed out if survey packages are not returned within two weeks, followed by a telephone call if the survey package is still not returned.

### Sample size estimation strategy and power analysis

The study will contact each patient referred for a surgical assessment for the surgical specialties included in Table 1. Precise estimates of procedures for power calculations are derive from historical utilization patterns. Based on surgical utilization statistics, there were at least 200 procedures conducted in 2010/2011 for each urology procedure in Table 1, providing 200 potential study patients per year for each procedure, noting that Vancouver General Hospital and St. Paul’s Hospital act as a provincial referral centre for a number of specialized procedures, while other specialties have similar, or greater, number of potential study patients.

This pool of recruits provides a sampling frame of 700 patients for each procedure (of the four year study design, we will recruit patients for 3.5 years). Among these, we expect that we will recruit approximately 48% of patients, leaving the study with 336 patients per procedure. Given our experience in similar studies, we expect to retain 70% of patients throughout the observation period, or observe complete data on 235 patients (equal to 700 times 0.48 times 0.70).

The assumed effect size of 0.2 SD is inferred from previous studies. To detect a treatment effect size over the W1 time of 0.2 standard deviation (SD) on decline in health, and probability of proceeding to surgical treatment, our study will require 220 patients, presuming alpha = 0.05 (two-tailed) and beta = 0.20 (power = 80%). Therefore, relative to our projected recruitment, our study is adequately powered to detect moderate sized effects in each surgery.

### Consent and protection of confidentiality in data

To ensure confidentiality of data, all study participants will be provided a unique study identifier which will be kept physically separate from patient-identifying information. A computer file matching the patient to their unique study identifier will be encrypted and kept on a separate hard disk. This study has been approved by UBC’s Behavioral Research Ethics Board (BREB).

### Measures

Table 2 provides an overview of the survey instruments that will be used in this proposed study. These instruments were selected because they are short, validated questionnaires that will not overly burden participants and have no or minimal licensing costs. We chose those instruments that provide a single score that can be compared across time (intra-participant comparison) and participants (inter-participant comparison). All instruments will be administered to the participant for the initial (baseline) survey. Subsequent surveys will exclude the co-morbidity questionnaire.

**Table 2:** **Patient reported outcome measure survey instruments and accompanying description**

Description	Instrument
**Generic assessment of health status**	EuroQol EQ-5D (5 L)
**Depression measure**	Patient Health Questionnaire (PHQ-9)
**Pain measure**	PEG-3
**Condition-specific assessment of health status**	Varies, depending on the procedure
**Assessment of medical and psychological syndromes**	Co-morbidity questionnaire
**Understanding of condition**	Knowledge questionnaire
**Sense of certainty with the treatment decision**	Decisional Conflict Scale

#### Generic assessment of health status

For the purposes of this study we use the EQ-5D-5 L to measure generic health status. The EQ-5D-5 L has been translated into 100 languages and is freely available to use, with no restrictions on publications. It is designed for self-completed post surveys and is easy to administer with minimal cognitive demands for study participants, taking only a few minutes to complete. The EQ-5D-5 L is widely used internationally and health states valuation norms have been validated in a Canadian population [19]. The EQ-5D-5 L has five questions addressing mobility, self-care, usual activities, pain/discomfort and anxiety/depression [19], which are scored using five levels. It also includes one vertical visual analogue scale on which the individual ranks their overall health status by making a mark along a line anchored by the endpoints labeled “best imaginable health state” and “worst imaginable health state”.

### Depression measure

To assess depression in patients’ we will use the Patient Health Questionnaire (PHQ-9). This instrument addresses both symptoms of depression and functional impairment [20]. There are nine questions regarding the presence of depression-related symptoms which are answered using a four-point Likert scale ranging from “not at all bothered” to “bothered nearly every day”.

### Pain measure

The PEG-3 will be used to assess pain in patients. The PEG has one intensity item and two interference items, for a total of three questions. Responses to the instrument’s three items are given a value between 0 and 10.

#### Assessment of medical and psychological syndromes

The research team has developed a questionnaire to assess the presence of common co-morbidities that may contribute to a patient’s clinical complexity. This questionnaire asks patients to report whether they have been diagnosed with common chronic, acute, or mental health conditions in the last three years. Responses to the questionnaire are not scored, rather they are used as a patient-level adjustment when analysing the study’s results.

#### Understanding of condition

A knowledge questionnaire asks patients about their understanding of their clinical problem, the treatment options available for that problem and the main benefits and potential side effects associated with those options. The knowledge questionnaires will be developed in conjunction with each of the participating specialities. Previous studies have generally developed a 6–8 item questionnaire using a multiple choice response format that have high degrees of internal consistency (alpha 0.82-0.83) and sensitivity to change [21–23]. These are scored based on the number of correct responses.

#### Certainty with treatment decision

The Decisional Conflict Scale will be used to assess a patient’s certainty about treatment. It is a validated, 16-item questionnaire [24]. Patients respond using a five item Likert scale, based on their level of agreement with a statement. High decisional conflict is associated with those patients who feel uninformed about their options, are unclear about their own personal values, or feel unsupported in making a decision.

### Preliminary data

To demonstrate the feasibility of recruiting patients into a study of this type and collecting patient-reported outcomes during the time that patients are waiting for surgical assessment, the study team conducted a pilot with orthopaedic surgeons in Vancouver. The study team worked with the surgeon’s staff to identify newly referred patients. A standardized script was prepared and used by the surgeon’s staff to summarize the study and describe the survey. Subsequently, patients were mailed a consent form and survey package. Completed packages were mailed to the surgeon’s office.

Fifty-five consecutive patients were contacted by the surgeon’s staff to determine whether they were willing to participate. Of those, three declined to participate and 52 surveys were mailed to patients. Of these patients, 27 ultimately returned the completed survey package with all assessment instruments completed, for a response rate of 48% (27 patients of 55 contacted).

The pilot of orthopaedic patients demonstrates the feasibility of collaborating with surgeon’s clinics to recruit patients newly referred to surgical assessment and to collect standardized patient health reported health, pain and depression measures during their wait.

## Discussion

There is an assumption in Canada that lengthy wait times for specialist consultations are an indicator of a poorly-performing healthcare system which can be resolved by expanding surgical supply or hospital capacity. In truth, there is little evidence regarding the effect of W1 on patients’ health and whether expanding surgical capacity would have any impact on outcomes. Given the investments federal and provincial stakeholders have made into improving access to elective surgical care, it is troubling that so little is known about the effects of waiting on patients’ health.

The conceptual model described above suggests that there is an opportunity to lower the W1 period. If there are specific characteristics that identify patients who are not certain of their decision to have surgery and are unlikely to proceed to surgery, yet still are on the wait list to be assessed by a surgeon, we could design algorithms to identify these patients in order to care for them in non-surgical ways. This would lower the wait time for those patients with appropriate referrals and improve the overall quality of healthcare by better matching treatments with patients’ preferences.

### Potential limitations

We are confident that this prospective longitudinal survey design is matched to meet the study’s objectives, but it has several limitations. These surveys suffer from sample attrition as patients drop out. Sample attrition is unavoidable in community-based studies, but through the use of reminders and communication with surgeons, we will take steps to mitigate attrition by making reminder calls and providing instructions to patients on how to update their contact information.

Self-selection bias is also a limitation of this survey design. We cannot be certain that those patients who opt out of the study do not systematically differ somehow from those that opt in. To address this we have allocated a significant budget to recruiting and following-up with participants.

Repeated questioning may cause participants to change their responses over time, a phenomena referred to as panel conditioning [25]. To mitigate this, we have minimized our survey points to every four months.

### Potential outcomes and future applications

This study will provide evidence to confirm or invalidate the theory that waiting for surgical assessment for elective surgeries is associated with negative consequences for patients’ health. If the results from this study do not indicate that waiting for elective surgical assessment has significant consequences for patients’ health, then this study challenges perceptions regarding wait times.

The knowledge gained from this study can be used to inform both policy-makers and clinicians regarding the health impact of waiting. For policy-makers, evidence from this study may form a foundation for decisions to brake (or accelerate) policy interventions designed to expedite surgical access. For clinicians, this study could identify opportunities to reduce inappropriate referrals for elective surgery either through better patient education interventions or improved shared decision-making.

## Abbreviations

BC: British Columbia
BREB: Behavioral Research Ethics Board
CIHI: Canadian Institute for Health Information
CIHR: Canadian Institutes for Health Research
GP: General Practitioner
UBC: University of British Columbia
VCH: Vancouver Coastal Health
W1: Wait one.

## References

[1] The Commonwealth Fund (2012). International Profiles of Health Care Systems, 2012.

[2] Prentice JC, Pizer SD (2007). Waiting times and hospitalizations for ambulatory care sensitive conditions. Heal Serv Outcomes Res Methodol.

[3] Prentice JC, Pizer SD (2007). Delayed access to health care and mortality. Health Serv Res.

[4] Ontario Medical Association (2011). OMA principles and recommendations: models and processes of delivery for specialty care.

[5] Badley EM, Canizares M, MacKay C, Mahomed NN, Davis AM (2013). Surgery or consultation: a population-based cohort study of use of orthopaedic surgeon services. Scherer RW, editor. PLoS One.

[6] Fitzgerald A, De Coster C, McMillan S, Naden R, Armstrong F, Barber A (2011). Relative urgency for referral from primary care to rheumatologists: the Priority Referral Score. Arthritis Care Res (Hoboken).

[7] Morris K, Zelmer J, Johnson T (2006). Wait times: a snapshot of what we know. Healthc Q.

[8] Fyie K, Frank C, Noseworthy T, Christiansen T, Marshall DA (2013). Evaluating the primary-to-specialist referral system for elective hip and knee arthroplasty. J Eval Clin Pract.

[9] Thind A, Stewart M, Manuel D, Freeman T, Terry A, Chevendra V (2012). What are wait times to see a specialist? An analysis of 26,942 referrals in Southwestern Ontario. Healthc Policy.

[10] Stainkey LA, Seidl IA, Johnson AJ, Tulloch GE, Pain T (2010). The challenge of long waiting lists: how we implemented a GP referral system for non-urgent specialist’ appointments at an Australian Public Hospital. BMC Health Serv Res.

[11] Wennberg JE, Fisher ES, Skinner JS (2002). Geography and the debate over medicare reform. Health Aff (Millwood).

[12] O’Connor AM, Llewellyn-Thomas HA, Flood AB (2004). Modifying unwarranted variations in health care: Shared decision making using patient decisions aids. Health Aff.

[13] Fraenkel L, Bogardus ST, Concato J, Wittink DR (2004). Treatment Options in Knee Osteoarthritis. Arch Intern Med.

[14] Sirovich B, Gallagher PM, Wennberg DE, Fisher ES (2008). Discretionary decision making by primary care physicians and the cost of U.S. Health care. Health Aff (Millwood).

[15] Robinson A, Thomson R (2001). Variability in patient preferences for participating in medical decision making: implication for the use of decision support tools. Qual Heal Care.

[16] Hack T, Degner L, Watson P, Sinha L (2006). Do patients benefit from participating in medical decision making? Longitudinal follow-up of women with breast cancer. Psychooncology.

[17] Dolan P (1996). The effect of experience of illness on health state valuations. J Clin Epidemiol.

[18] Pereira C, Palta M, Mullahy J, Fryback D (2011). Race and preference-based health-related quality of life measures in the United States. Qual Life Res.

[19] EuroQoL Group (2011). EQ-5D.

[20] Löwe B, Unützer J, Callahan CM, Perkins AJ, Kroenke K (2004). Monitoring depression treatment outcomes with the patient health questionnaire-9. Med Care.

[21] Stacey D, O’Connor AM, DeGrasse C, Verma S (2003). Development and evaluation of a breast cancer prevention decision aid for higher-risk women. Heal Expect.

[22] Man-Son-Hing M (1999). A patient decision aid regarding antithrombotic therapy for stroke prevention in atrial fibrillation. A randomized controlled trial. JAMA.

[23] O’Connor AM, Tugwell P, Wells GA, Elmslie T, Jolly E, Hollingworth G (1998). A decision aid for women considering hormone therapy after menopause: decision support framework and evaluation. Patient Educ Couns.

[24] O’Connor AM (1995). Validation of a decisional conflict scale. Med Decis Mak.

[25] Sturgis P, Allum N, Brunton-Smith I, Lynn P (2009). Attitudes over time: the psychology of panel conditioning. Methodology of longitudinal surveys.

[CR26] The pre-publication history for this paper can be accessed here: http://www.biomedcentral.com/1471-2482/15/4/prepub

